# The Health and Well-Being of African-American Older Adults With a History of Incarceration

**DOI:** 10.1093/geroni/igaa057.1633

**Published:** 2020-12-16

**Authors:** Rodlescia Sneed

**Affiliations:** Michigan State University, Flint, Michigan, United States

## Abstract

African-Americans are overrepresented in the criminal justice system. Longer prison stays and release programs for older prisoners may result in an increased number of community-dwelling older adults with a history of incarceration. In recent years, there has been a substantial increase in research on health-related outcomes for currently incarcerated older adults; however, there has been little inquiry into outcomes for formerly incarcerated African-American older adults following community re-entry. In this study, we used secondary data from the Health and Retirement Study to describe employment, economic, and health-related outcomes in this population. Twelve percent of the 2238 African-Americans in our sample had been previously incarcerated. Those who had been previously incarcerated had higher rates of lung disease, arthritis, back problems, mobility problems, and mental health issues than their counterparts. They also had higher rates of hospitalization and lower use of dental health services. Further, while they did not experience lower employment rates than those with no criminal history, those who had been incarcerated had more physically demanding jobs and reported greater economic strain. Given the disproportionate incarceration rates among African-Americans, the aging of the prison population, and the increase in community re-entry for older prisoners, research that explores factors that impact the health and well-being of formerly incarcerated individuals has broad impact. Future work should focus on addressing the needs of this vulnerable population of African-American older adults.

